# Effect of Anesthesia Applied for Magnetic Resonance Imaging on the Body Temperature of Pediatric Patients

**DOI:** 10.7759/cureus.5705

**Published:** 2019-09-20

**Authors:** Öznur Uludağ, Recai Kaya, Atilla Tutak, Mevlüt Doğukan, Mustafa Çelik, Ebru Dumlupınar

**Affiliations:** 1 Anesthesiology and Reanimation, Adıyaman University Faculty of Medicine, Adıyaman, TUR; 2 Anesthesiology and Reanimation, Private Hospital, Osmaniye, TUR; 3 Anesthesiology and Reanimation, Adıyaman University Faculty of Medicine, Adiyaman, TUR; 4 Psychiatry, Private Hospital, Gaziantep, TUR; 5 Biostatistics and Medical Informatics, Adıyaman University Faculty of Medicine, Adiyaman, TUR

**Keywords:** magnetic resonance imaging, anesthesia, temperature change, pediatric patients

## Abstract

Objective

Anesthesia may be required to ensure the immobility of the patient during a magnetic resonance imaging (MRI) scan, particularly in pediatric patients. An MRI scanner generates radiofrequency radiation (RFR) to obtain images of parts of the body. During an MRI procedure, an amount of RFR is transformed into heat by the body, thereby leading to increased body temperature. However, patients are at increased risk of hypothermia due to the impairment of thermoregulation by anesthesia and the cold and dry environment of the MRI room. The aim of this study was to investigate the effects of anesthesia on body temperature with regard to patient safety in pediatric patients undergoing an MRI scan.

Materials and methods

The study included a total of 40 children aged three to 10 years who underwent an MRI procedure. The patients were divided into two groups based on the administration of anesthesia: (I) non-sedated and (II) sedated. Prior to the procedure, non-sedated patients were informed about the procedure by a psychiatrist. Body temperature was measured from the tympanic membrane and skin in each patient. The MRI scan was performed at room temperature (20°C-22°C) with a relative humidity of 35%-40%.

Results

No significant change was found between pre- and post-scan body temperatures in Group I, whereas a significant decrease was found between pre- and post-scan body temperatures in Group II. No complication occurred in any patient due to temperature change or anesthesia.

Conclusion

A significant decrease in body temperature was found in pediatric patients undergoing an MRI procedure under sedation. The results implicated that anesthesia has a remarkable effect on the balance between the temperature increase caused by RFR and the temperature decrease caused by anesthesia.

## Introduction

Magnetic resonance imaging (MRI) is accepted as a prevalent imaging modality worldwide [1]. It has been reported that the number of pediatric patients undergoing an MRI or computed tomography (CT) scan increases by 8%-9% per year [2]. Patients undergoing MRI can experience a wide range of emotions such as uncontrollable fear, slight nervousness, and anxiety. Children, in particular, are highly likely to experience anxiety and distress during an MRI procedure due to various reasons, including the confined space and noise of the scanner. The scanning procedure often needs to be repeated due to movement artifacts, which result in increased radiation exposure and healthcare costs [3]. Sedation or general anesthesia is required in pediatric patients undergoing an MRI procedure and absolute immobility is required to obtain ideal images. Therefore, anesthetic management of the patient is of paramount importance in terms of patient safety.

An MRI scanner generates radiofrequency radiation (RFR) to obtain images of parts of the body. When the patient is placed in a strong magnetic field, such as from an MRI scanner, the axes of protons all line up and become ready to be warned. When a radio wave is sent to the body, these protons deviate from their position by the energy they receive. When the RFR ends, the protons emit the energy they receive. MRI images are obtained by processing these energies. An amount of RFR is transformed into heat by the body [4]. The quantity of RFR absorbed during an MRI examination is described as the specific absorption rate. On the other hand, the duration of exposure to RFR affects thermoregulatory changes and an increase in body temperature is expected during the MRI procedure. Additionally, in pediatric patients, anesthetic agents are administered to ensure the immobilization of the patient throughout the procedure and these agents affect the thermoregulatory mechanisms. It is also known that sedated children may develop anesthesia-induced hypothermia [5]. Moreover, the cool and dry environment of the MRI room may also lead to a temperature change.

The present study aimed to examine the effects of anesthesia on body temperature with regard to patient safety in pediatric patients undergoing an MRI scan.

## Materials and methods

The study protocol was approved by the Adiyaman University Ethics Committee (Approval Date: 25.12.2014, No: 57831858/105) and was performed in accordance with the 1964 Helsinki Declaration and its later amendments. The study was designed as a prospective observational case series study and included a total of 40 patients based on the sample size calculated for a power of 95% and a significance level of 0.05, as proposed in previous studies. All the patients were aged from three to 10 years and had an American Society of Anesthesiologists (ASA) score of 1-2.

The exclusion criteria were ASA score >2, severe pulmonary or cardiovascular disease, anatomic airway abnormalities, and baseline body temperature ≥37.5 °C. Each patient was evaluated one week prior to the MRI procedure and informed consent was obtained from the parents of each patient. The parents were advised to apply an appropriate overnight fasting period prior to the procedure. All the patients were requested to wear the same type of hospital coat throughout the procedure. No contrast agent was used in any patient. The routine MRI protocol was administered at room temperature (20°C-22°C) with relative humidity (35%-40%) using a 1.5 T MRI device (Philips Achieva, Netherlands). The same temperature was maintained and was continuously measured by an anesthetist (Ö.U.) throughout the procedure. The patients were divided into two groups based on the administration of anesthesia: (I) non-sedated and (II) sedated. Prior to the procedure, all non-sedated patients were informed about the procedure by a psychiatrist (M.C.). The patients in Group II received midazolam 0.1 mg kg^-1^ intravenously (i.v.) prior to the procedure while the patients in Group I received no anesthesia. The patients were then taken to the MRI room and the anesthesia personnel remained in the MRI room throughout the procedure. In Group II, anesthetic induction was performed with propofol 1 mg kg i.v. and an additional dose of propofol 0.5 mg kg^-1^ was given as needed. Oxygen was delivered with a pediatric face mask with a gas flow rate of 2 l/min^−1^. A shoulder roll was placed behind the neck to prevent airway problems. Data were recorded by an independent observer. An MRI-compatible anesthetic device was used for anesthesia. Continuous electrocardiogram (ECG), peripheral capillary oxygen saturation (SpO2), and respiratory rate monitoring were performed with an MRI-compatible monitor throughout anesthesia. Body temperature was measured from the skin and the same tympanic membrane in each patient. Age, weight, duration of MRI procedure, and total time spent in the MRI room were recorded. At the end of the scan, the patients were transferred to the post-anesthesia care unit (PACU) and were kept there until they were fully awake and performed intentional movements.

Statistical analysis

Data analysis was performed using SPSS for Windows version 22.0 (IBM SPSS Inc. Co., Armonk, NY, USA). Normal distribution of data was analyzed using the Kolmogorov-Smirnov test. A paired t-test was used for the comparison of two normally distributed dependent groups and the Wilcoxon test was used for the comparison of two non-normally distributed dependent groups. Continuous variables were expressed as mean ± standard deviation (SD) or mean (minimum-maximum) based on their distribution pattern. Categorical variables were expressed as frequencies (n) and percentages (%). A p-value of <0.05 was considered significant.

## Results

The patients comprised 24 (60%) men and 16 (40%) women with a mean age of 4.72 ±1.51 years and a mean bodyweight of 17.52±4.5 kg. The mean duration of the MRI procedure was 15 (range, 10-60) min, and the mean total time spent in the MRI room was 20 (range, 15-65) min (Table 1).

**Table 1:** Demographic and clinical data

		n=40	
Male	24 (60%)	
Female	16 (40%)	
Age (years)		4.72 ± 1.51	
Weight (kg)		17.52 ± 4.5	
MRI procedure duration (minutes) (min-max)		15 (10-60)	
Total duration (minutes) (min-max)		20 (15-65)	

No significant change was found between pre- and post-scan tympanic temperatures in Group I (37.01±0.319 and 36.98±0.63°C, respectively) (p=0.810). However, a significant decrease was found between pre- and post-scan tympanic temperatures in Group II (36.65±0.34 and 36.13±0.44°C, respectively) (p˂0.05). Similarly, although no significant change was found between pre- and post-scan skin temperatures in Group I (36.59±0.39 and 36.62±0.65°C, respectively) (p=0.766), a significant decrease was found between pre- and post-scan skin temperatures in Group II (36.41±0.35 and 35.97±0.49°C, respectively) (p˂0.05) (Table 2).

**Table 2:** Temperature measurements

		Group I (Non-Sedated)	Group II (Sedated)	
Tympanic temperature	Pre-MRI	37.01 ± 0.319	36.65 ± 0.34
	Post-MRI	36.98 ± 0.63	36.13 ± 0.44	
	p	0.810	<0.05	
Skin temperature	Pre-MRI	36.59 ± 0.39	36.41 ± 0.35
	Post-MRI	36.62 ± 0.65	35.97 ± 0.49	
	p	0.766	<0.05	

Table 3 presents the changes in tympanic and skin temperatures. No complication occurred in any patient due to temperature change or anesthesia.

**Table 3:** Changes in tympanic and skin temperatures

	Tympanic Temperature	Skin Temperature
Pre-MRI (°C)	36.83 ± 0.37	36.50 ± 0.38
Post-MRI (°C)	36.56 ± 0.68	36.29 ± 0.66
p	0.002	0.009
Temperature Change (°C)	0.7 ± 1.54	0.62 ± 1.54
Decreased	n=25 (62.5%) 0.96 ± 1.91	n=26 (65%) 0.82 ± 1.92
Increased	n=12 (%30) 0.33 ± 0.26	n=12 (%30) 0.32 ± 0.28
No Change	n=3 (7.5%)	n=2 (5%)

The quality of MRI images was evaluated by a single radiologist, and it was revealed that the image quality was remarkably good.

## Discussion

Anesthetic drugs disrupt thermoregulation in all age groups. However, children are more susceptible to hypothermia, as their skin is thinner and they are less capable of controlling cold stress as compared to adults [6]. Meaningfully, when children are exposed to cold and dry conditions during an MRI procedure, their body temperature is likely to decrease. A previous study evaluated human thermal response to radiation-induced heating during an MRI procedure and found no excessive temperature elevations or other deleterious physiological consequences related to radiation exposure [7]. Another study, however, evaluated patients who underwent brain MRI under sedation and revealed that the patients had an average temperature increase of 0.5°C between pre- and post-scan measurements [8]. In contrast, Tsui et al. reported that patients had an average temperature decrease of 0.3°C in children that underwent MRI under sedation [5]. Another study evaluated the temperature differences between two MRI devices (1.5T and 3T) in patients who underwent propofol infusion following midazolam premedication. The authors reported that body temperature was increased by both devices, with an average increase of 0.2°C on 1.5T and 0.5°C on 3T MRI units. Additionally, the authors measured both tympanic and rectal temperatures and revealed that the temperature measurements were similar between the right tympanic and rectal sites both before and after the scans. The authors concluded that the tympanic temperature accurately reflects rectal temperature measurements and that body temperature increases as the magnetic field strength of the MRI device increases [9].

Lo et al. evaluated temperature changes in pediatric patients undergoing anesthesia for an MRI procedure and reported a temperature decrease ranging from -0.36°C to -0.19°C in the patients. The authors suggested that the cooling effects of anesthetic agents during an MRI scan rather than the warming effects of the MRI scanner should be considered [10]. Another study used a 3T MRI device in newborns undergoing an MRI procedure under sedation and found no hyperthermic changes in any patient [11]. In contrast, Don et al. evaluated children undergoing MRI and reported that the risk of hypothermia was the highest during the MRI scan [12]. Isaacson et al. measured temporal artery temperature in children undergoing MRI exams on 1.5T and 3T MRI units and found a correlation between sedation and body weight, age. Nevertheless, no clinically significant core body temperature change was detected in any patient [7]. In our study, we measured both tympanic and skin temperatures to obtain more reliable results, and it was revealed that body temperature decreased in the sedated group during the MRI scan. It is commonly known that when the cause of warming is confronted particularly with RF propagation, certain drugs may have an inverse synergistic effect in terms of tissue warming. It has also been estimated that even in an applied field of 1 T, the free energy barrier to dissociation is changed by only 1 J/mol. This energy exchange has less effect on the reaction balance as compared to a temperature change of 0.01°C [13].

## Conclusions

The results implicated that hypothermia should be considered in patients receiving anesthesia. Although MRI-compatible temperature-measurement systems are highly expensive and used scarcely due to the overwhelming effect of the magnetic field, we believe that routine temperature monitoring is highly important for the safety of pediatric patients undergoing MRI and will also improve the quality of the MRI procedure in terms of patient safety.

## References

[1] Hallowell LM, Stewart SE, De Amorim ESCT, Ditchfield MR (2008). Reviewing the process of preparing children for MRI. Pediatr Radiol.

[2] Wachtel RE, Dexter F, Dow AJ (2009). Growth rates in pediatric diagnostic imaging and sedation. Anesth Analg.

[3] Arlachov Y, Ganatra RH (2018). Sedation/anaesthesia in paediatric radiology. Br J Radiol.

[4] Rodriguez AO (2004). Principles of magnetic resonance imaging [Article in Spanish]. Rev Mex Fis.

[5] Tsui BC, Wagner A, Usher AG, Cave DA, Tang C (2005). Combined propofol and remifentanil intravenous anesthesia for pediatric patients undergoing magnetic resonance imaging. Pediatr Anaesth.

[6] Cote CJ (2010). Pediatric Anesthesia. https://www.elsevier.com/books/millers-anesthesia-2-volume-set/miller/978-1-4557-0876-5.

[7] Isaacson DL, Yanosky DJ, Jones RA, Dennehy N, Spandorfer P, Baxter AL (2011). Effect of MRI strength and propofol sedation on pediatric core temperature change. J Magn Reson Imaging.

[8] Bryan YF, Templeton TW, Nick TG, Szafran M, Tung A (2006). Brain magnetic resonance imaging increases core body temperature in sedated children. Anesth Analg.

[9] Machata AM, Willschke H, Kabon B, Prayer D, Marhofer P (2009). Effect of brain magnetic resonance imaging on body core temperature in sedated infants and children. Br J Anaesth.

[10] Lo C, Ormond G, McDougall R, Sheppard SJ, Davidson AJ (2014). Effect of magnetic resonance imaging on core body temperature in anaesthetised children. Anaesth Intensive Care.

[11] Cawley P, Few K, Greenwood R (2016). Does magnetic resonance brain scanning at 3.0 Tesla pose a hyperthermic challenge to term neonates?. J Pediatr.

[12] Don Paul JM, Perkins EJ, Pereira-Fantini PM (2018). Surgery and magnetic resonance imaging increase the risk of hypothermia in infants. J Paediatr Child Health.

[13] Schenck JF (1992). Health and physiological effects of human exposure to whole-body 4 Tesla magnetic fields during MRI. Ann NY Acad Sci.

